# *Notes from the Field:* Assessment of State-Level Influenza Season Severity — Minnesota and Utah, 2017–18 Influenza Season

**DOI:** 10.15585/mmwr.mm6806a7

**Published:** 2019-02-15

**Authors:** Michelle M. Hughes, Joshua D. Doyle, Keegan McCaffrey, Melissa McMahon, Melanie Spencer, Karen Martin, Gregg M. Reed, Anna E. Carmack, Shikha Garg, Melissa Rolfes, Carrie Reed, Matthew Biggerstaff

**Affiliations:** ^1^Epidemic Intelligence Service, CDC; ^2^Influenza Division, National Center for Immunization and Respiratory Diseases, CDC; ^3^Utah Department of Health; ^4^Minnesota Department of Health, ^5^Salt Lake County Health Department, Salt Lake City, Utah; ^6^University of Maryland Medical Center, Baltimore, Maryland.

The U.S. 2017–18 influenza season was a high-severity season, with the highest number of outpatient visits for influenza-like illness* (ILI) since the 2009–10 pandemic and the highest rate of influenza-associated hospitalizations since surveillance expanded to include adult hospitalizations during the 2005–06 season (*1*). The severe season was characterized by reports of strained emergency departments and hospitals and spot shortages of influenza antiviral medications (*2*). Influenza activity can vary widely across geographic regions (*3*), and local severity assessments might better guide public health actions and health care needs and support the development of tailored communication messages to prevent influenza morbidity and mortality. CDC assesses influenza season severity at the national level (*4*),^†^ but the applicability of this approach at state or local levels has not been tested.

In February 2018, field investigations were conducted in Minnesota and Utah to identify potential indicators of state-level influenza activity and pilot a state-level approach to assessing influenza season severity in real time. Indicators were selected using three criteria: 1) availability of data for 2017–18 and at least five previous influenza seasons; 2) completeness and representativeness of data on observed influenza seasonality; and 3) timeliness. Two indicators selected in both states were weekly ILI activity (percentage of outpatient visits to sentinel providers for ILI) and influenza-associated hospitalizations (counts or population-based rates). A third indicator included weekly counts of influenza-associated deaths in Minnesota and weekly percentage of specimens testing positive for influenza reported by sentinel clinical laboratories in Utah. Using state-level data from five earlier seasons (2012–13 through 2016–17) and following previously published procedures (*3*), indicator-specific intensity thresholds (ITs) for a 50% chance (IT_50_), 10% chance (IT_90_), and a 2% chance (IT_98_) of observing higher values during the 2017–18 season were calculated. Severity was classified as low, moderate, high, or very high if at least two of three indicators peaked during the 2017–18 season below their IT_50_ value, between their IT_50_ and IT_90_ values, between their IT_90_ and IT_98_ values, and above their IT_98_ value, respectively.

The interim severity of the 2017–18 influenza season (assessed in mid-February 2018) for both Minnesota and Utah was categorized as high. As an example of one of the three indicators, influenza-associated hospitalizations through the end of the 2017–18 season (May 2018) peaked above the IT_90_ (Minnesota) and IT_98_ (Utah) values (Figure). End-of-season severity assessments for both states remained high, aligning with national trends and the subsequent high severity classification for the entire United States (*1*).

**FIGURE:**
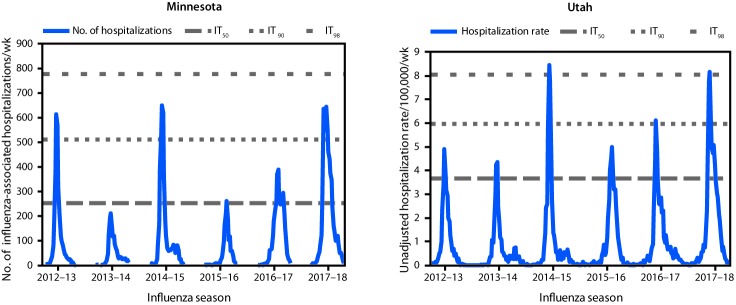
Influenza-associated hospitalizations indicators and intensity thresholds — Minnesota*^,^^†^ and Utah,^§,¶^ 2012–18 influenza seasons **Abbreviations:** IT_50_ = intensity threshold at which there is a 50% chance of observing a higher value during 2017–18 based on historical (2012–13 through 2016–17) peak values; IT_90_ = intensity threshold at which there is a 10% chance of observing a higher value during 2017–18 based on historical (2012–13 through 2016–17) peak values; IT_98_ = intensity threshold at which there is a 2% chance of observing a higher value during 2017–18 based on historical (2012–13 through 2016–17) peak values. * Reported to the Minnesota Department of Health. ^†^ Minnesota intensity thresholds: IT_50_ = 255; IT_90_ = 511; IT_98_ = 778. ^§^ Reported to the Utah Department of Health. ^¶^ Utah intensity thresholds: IT_50_ = 3.66; IT_90_ = 5.99; IT_98_ = 8.06.

The national severity assessment framework was successfully adapted for use in Minnesota and Utah. Utah is piloting the report of the weekly severity assessments for the 2018–19 season (*5*). Additional states might find this method useful for improving local public health messaging, preparedness, and response during an influenza season and in the event of a pandemic. CDC continues to develop resources to support local assessments of influenza season severity; interested jurisdictions are encouraged to contact CDC’s Influenza Division for assistance.

## Footnotes

* Fever (temperature ≥100°F [37.8°C]) and a cough and/or a sore throat without a known cause other than influenza.

† Nationally, CDC assesses flu severity using three indicators: 1) percentage of visits to outpatient clinics for ILI; 2) the rates of influenza-associated hospitalizations; and 3) the percentage of deaths resulting from pneumonia or influenza that occurred during each season.
